# Can probiotics, prebiotics and synbiotics improve functional outcomes for older people: a systematic review

**DOI:** 10.1007/s41999-020-00396-x

**Published:** 2020-09-24

**Authors:** L. Coutts, K. Ibrahim, Q. Y. Tan, S. E. R. Lim, N. J. Cox, H. C. Roberts

**Affiliations:** Academic Geriatric Medicine, Southampton General Hospital Mailpoint 807, University of Southampton, Southampton, SO16 6YD UK

**Keywords:** Probiotics, Prebiotics, Synbiotics, Older, Functional outcomes

## Abstract

**Purpose:**

Research evaluating the effect of probiotics, prebiotics and synbiotics (PPS) on laboratory markers of health (such as immunomodulatory and microbiota changes) is growing but it is unclear whether these markers translate to improved functional outcomes in the older population. This systematic review evaluates the effect of PPS on functional outcomes in older people.

**Methods:**

We conducted a systematic review of the effect of PPS in older adults on functional outcomes (physical strength, frailty, mood and cognition, mortality and receipt of care). Four electronic databases were searched for studies published since year 2000.

**Results:**

Eighteen studies (including 15 RCTs) were identified. One of five studies evaluating physical function reported benefit (improved grip strength). Two analyses of one prebiotic RCT assessed frailty by different methods with mixed results. Four studies evaluated mood with no benefit reported. Six studies evaluated cognition: four reported cognitive improvement in participants with pre-existing cognitive impairment receiving probiotics. Seven studies reported mortality as a secondary outcome with a trend to reduction in only one. Five studies reported length of hospital stay but only two peri-operative studies reported shorter stays.

**Conclusion:**

There is limited evidence that probiotics may improve cognition in older people with pre-existing cognitive impairment but no clear evidence of benefit of PPS on physical function, frailty, mood, length of hospitalisation and mortality. Larger studies with more homogenous interventions, accounting for confounding factors, such as diet, co-morbidities and medications, are required. There is currently inadequate evidence to recommend PPS use to older people in general.

**PROSPERO registration number:**

PROSPERO registration number is CRD42020173417. Date of PROSPERO registration: 01/05/20.

**Electronic supplementary material:**

The online version of this article (10.1007/s41999-020-00396-x) contains supplementary material, which is available to authorized users.

## Key summary points

**Aim:**

To review current evidence on whether probiotics, prebiotics and synbiotics improve functional outcomes for older people.

**Findings:**

There is limited evidence that probiotics might improve cognition in older people with pre-existing cognitive impairment. There is little evidence for benefit of probiotics, prebiotics and synbiotics on physical function, frailty, mood, mortality or length of hospital admission among older people, although the 18 studies identified for the review were heterogeneous and these functional outcomes were largely secondary outcomes.

**Message:**

More robust research with larger studies, consistency of interventions and clear assessment of confounding variables (such as diet, co-morbidities and medications) is needed to evaluate the effect of probiotics, prebiotics and synbiotics on functional outcomes in an older population.

**Electronic supplementary material:**

The online version of this article (10.1007/s41999-020-00396-x) contains supplementary material, which is available to authorized users.

## Background

There has been considerable focus on the role of the human microbiome on health since the human microbiome project launched in the United States in 2007 [1]. Within this, there is a growing field of interest in the potential role of probiotics, prebiotics and synbiotics (PPS) in altering the gastrointestinal microbiome for health benefits. These interventions appear to be a relatively safe and cheap option for healthcare providers. A growing commercial industry in this area provides a range of formulas that claim to improve the consumers’ well-being [2]. Many older people may buy these products expecting to improve their health [3]; however, their effects on the health of older people have not been well studied.

A probiotic is briefly defined as bacteria (e.g. *Lactobacillus*) or other microorganisms recognised to be beneficial to the human body and occur in or are added to certain foods and supplements [4]. They are widely available in yoghurts, drinks or supplements in the form of tablets, capsules or powder. The concept is that ‘healthy bacteria’ are added into the intestinal microbiome. *Lactobacillus* and *Bifidobacterium* species are most commonly used. Thousands of different strains exist within these species.

Prebiotics are defined as “nondigestible constituents of food, such as inulin and fructo-oligosaccharides, which stimulate the growth of ‘good’ bacteria in the colon” [4]. The concept of prebiotics is that they nurture the ‘healthy’ bacteria already in the gastrointestinal tract and so promote further growth of these selected types of bacteria rather than directly trying to administer them (as with probiotics). As with probiotics, the main targets are *Bifidobacterium* and *Lactobacillus *species. Prebiotics can be difficult to define as they can take many forms and are often naturally occurring. Some fruit and vegetables can have prebiotic properties (including Jerusalem artichoke, onions, chicory, leeks, asparagus and bananas). Commercial or medicalised prebiotics generally contain oligosaccharides. The commonly used laxative, Lactulose, can also act as a prebiotic as it is anaerobically fermented in the colon by the microbiota and serves as a prebiotic substrate by increasing the count of *Bifidobacterium,* *Lactobacillus* and bacterial metabolites like short-chain fatty acids [5]. The fact that there is extensive inter-individual variation in gut microbiota composition suggests that there may be inter-individual variation in the response of microbial communities to prebiotics too [6].

Synbiotics are a combination of probiotics and prebiotics that act synergistically to improve gastrointestinal health. These commonly take the form of capsules, tablets or powders and normally contain a combination of bacteria and oligosaccharides.

Few studies evaluating the impact of PPS have focused on the older population living with frailty. Gut microbiota change significantly with age with an increase in proteolytic bacteria and a decrease in saccharolytic bacteria [7] and so findings from studies of younger cohorts may not apply to older people. Age-related changes in diet, digestion, transit time, colonic pH and salivary function all affect the intestinal microbiota [8]. Studies show that a less diverse intestinal microbiome has been associated with frailty [9] and that people surviving beyond 99 years of age have a higher diversity of gut microbiota and more ‘health-related species’ compared to the general older population [10].

Much of the research into the effect of PPS to date has focused on cellular biology. The hypothesis of a ‘leaky gut’ being associated with an unhealthy intestinal microbiome and leading to chronic inflammation (‘inflammaging’) has led to many studies evaluating the links between bacteria in the gastrointestinal tract and immunomodulatory markers [11, 12]. Studies report evidence of beneficial immunomodulatory changes in older people taking PPS [13] and beneficial changes to their intestinal microbiome [14], but it is unclear whether these laboratory findings translate into functional outcomes relevant to both clinicians and older people themselves.

This systematic review will evaluate current evidence on the effect of PPS on functional outcomes relevant to older people and clinicians: physical function, frailty, mood, cognition, mortality and receipt of care.

## Methods

We conducted a systematic review of all studies using the Preferred Reporting Items for Systematic Reviews and Meta-Analysis (PRISMA) approach. The protocol was registered on the PROSPERO database: registration number: CRD42020173417.

A systematic search of the literature was conducted in four electronic databases: Medline, EMBASE, CINAHL and Pubmed. The search strategy reflected the PICOS elements (Table 1) and is reported in Appendix 1.

**Table 1:** PICOS elements

Population	Mean age ≥ 65 years
Intervention	Single intervention of probiotic, prebiotic or synbiotic
Comparison	Placobo or no intervention
Outcome	1. Physical function 2. Frailty 3. Mood and cognition 4. Mortality 5. Receipt of care- hospital admissions or length of stay
Study design	Any design or setting

Inclusion criteria were studies of older adults (defined as minimum mean age of 65 years) published in the last 20 years (since year 2000). We excluded studies evaluating multiple interventions (such as exercise paired with a PPS). Grey literatures, such as theses or commentary articles, were excluded. No language or geographical limitations were applied. Where non-English studies were identified that met our inclusion criteria, attempts were made to translate the publications prior to data extraction and analysis. Studies were excluded from the final analysis if we were unable to achieve translations.

A single reviewer (LC) screened the study titles for relevance. Two reviewers (LC, KI) then independently screened the remaining abstracts. Full-text articles were assessed for eligibility by two reviewers again working independently (LC, KI/QYT). Reasons for exclusion were documented and any disagreements were discussed and reviewed with the senior author.

LC extracted data from eligible studies. Two independent reviewers then conducted quality appraisal of the included studies using the Critical Appraisal Skill Programme tool and graded the quality of the papers using GRADE certainty criteria [15] (LC, SERL/NJC). The GRADE method assesses certainty of evidence specific to a question considering potential for bias, imprecision, inconsistency, indirectness and publication bias.

The included studies were heterogenous and so a meta-analysis of outcomes was not possible. Instead a narrative analysis was conducted for each functional outcome listed in Table 1.

## Results

**Fig. 1:**
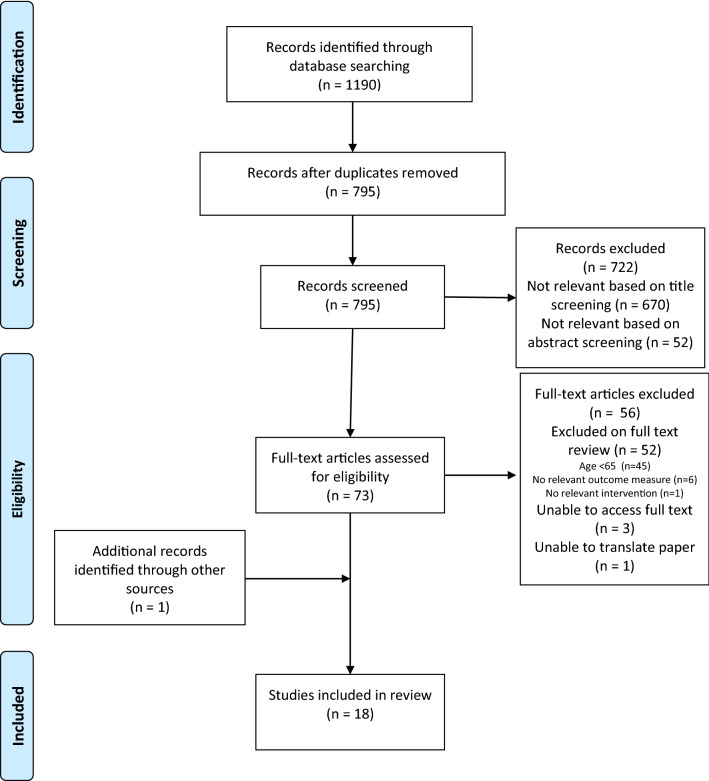
PRISMA flow diagram

This review identified 15 randomised controlled trials (RCTs), two secondary analyses of previous RCTs and one single-arm trial (18 papers in total). Ten studies used a probiotic as intervention, three used a prebiotic and five used a synbiotic. Seven were conducted in hospital settings, seven in the community, three in nursing homes and one in a welfare organisation. The total number of participants across the 18 studies was 1803 with sample size ranging from 17 to 278 participants. None of the interventions used were directly comparable; two probiotic interventions contained the same bacteria, but at different strengths and two synbiotic interventions used the same product but at different doses, otherwise there were no matched interventions. We report the findings for each functional outcome: physical function, frailty, cognition and mood, mortality and length of hospital admission. ‘Receipt of care’ was limited to length of hospital admission as no studies reporting number of hospital admissions were identified (Fig. 1; Table 2).

**Table 2:** Study characteristics and data extraction

Study	Mean age (SD) [range] unless otherwise stated	Participants		Setting	Study type	Intervention	Comparator	Duration of intervention	Outcome findings relevant to our systematic review (intervention vs. control) mean (SD) unless otherwise stated	GRADE certainty
		No	Characteristics							
Akbari et al. [24]	Control: 82 (± 1.7) Intervention: 77.7 (± 2.6)	60	People with Alzheimer’s dementia	Welfare organisations, Iran	RCT	PROBIOTIC (+ Milk) *Lactobacillus acidophilus, Lactobacillus casei, Bifidobacterium bifidum, Lactobacillus fermentum* (2 × 10^9^ CFU/g of each) 200 ml/day	Milk (200 ml/day)	12 weeks	Cognition: Improved MMSE with intervention + 27.90% (± 8.07) vs − 5.03% (± 3.00) (*p* < 0.001)	Low
Hwang et al. [25]	Control: 69.2 (± 7.0) Intervention: 68.0 (± 5.1)	100	Individuals with mild cognitive impairment	Community, South Korea	RCT	PROBIOTIC *Lactobacillus plantarum *C29 (from kimchi) (≥ 1.25 × 10^10^ CFU/g) Fermented soybean (DW2009) 62.5% fermented soybean 800 mg/day	Placebo capsules (cellulose)	12 weeks	Cognition: Improved combined cognitive function with intervention and specifically improved attention domain. No significant improvement in working or verbal memory domains Combine cognitive function: 0.95 to 0.79 vs. 0.68 to 0.65 (*p* for interaction = 0.02) Attention composite score: 1.44 to 1.05 vs. 0.82 to 0.70 (*p* for interaction = 0.02) Working memory composite score 1.12 to 1.08 vs. 0.98 to 1.06 (*p* for interaction = 0.63) Verbal memory composite score 0.99 to 0.98 vs. 0.87 to 0.93 (*p* for interaction = 0.21	Low
Kobayashi et al. [26]	All: 82.5 (± 5.3)	27	Older persons attending monthly outpatient rehab with mild cognitive impairment	Community, Japan	Pilot Single arm study	PROBIOTIC *Bifidobacterium breve *A1 (~ 2 × 10^10^ CFU) Two capsules after meals	Nil	24 weeks	Cognition: Improved cognition with intervention MMSE after 24 weeks + 1.7 (*p* < 0.01) Serial 7 s after 24 weeks + 1.1 (*p* < 0.01) ‘Resolution’ of MCI in 13/19 cases by 24 weeks DSST after 24 weeks + 0.74 (*p* = 3.2)—no significant change Mood: No change to mood No significant change to TMD scores and subscales	Very low
Kotzampassi et al. [36]	Control: 66.4 (± 11.9) Intervention: 65.9 (± 11.5)	168	All adult patients consecutively admitted for elective colonic resection with primary anastomosis	Surgical unit, Greece	RCT	PROBIOTIC *Lactobacillus acidophilus* LA-5 (1.75 × 10^9^ CFU),* Lactobacillus plantarum *(0.5 × 10^9^ CFU),* Bifidobacterium lactis *BB-12 (1.75 × 10^9^ CFU), *Saccaromyces boulardii *(1.5 × 10^9^ CFU) Four capsules preoperatively then one capsule BD for next 14 days with 100 ml water	Placebo capsules (powdered glucose polymer)	14 days	Length of stay: Reduced length of stay postoperatively with intervention Median time until discharge: 8 days vs. 10 days (log-rank: 20.30, *p* < 0.0001)	Very low
Mañé et al. [20]	Control: Median age: 69 [IQR: 66–82] Intervention 1: Median age: 70 [IQR: 67–83] Intervention 2: Median age: 71 [IQR: 65–84]	50	Older participants (> 65) institutionalised in one of two centres	Institutional geriatric centres, Spain	RCT	PROBIOTIC + milk powder *Lactobacillus plantarum *CECT 7315 and CECT 7316 Low dose: 5 × 10^8^ CFU/day in 20 g of powdered skimmed milk High dose: 5 × 10^9^ CFU/day in 20 g of powdered skimmed milk	Placebo (20 g of powdered skimmed milk)	12 weeks	Mortality: Trend towards positive effect on survival but underpowered study Mortality during treatment: low dose probiotic 0/13, High dose probiotic 0/19, Placebo 3/18 Physical function: No significant change in ADLs demonstrated No significant change in Barthel index (follow up data not shown)	Low
McNaught et al. [32]	Control: Median age: 71 [IQR: 65–77] [28–90] Intervention: Median age: 71 [IQR: 60–77] [28–87]	103	Adults admitted to ITU	Intensive Care Unit, UK	RCT	PROBIOTIC + conventional therapy Proviva—oatmeal and fruit drink containing *Lactobacillus plantarum *299v (5 × 10^7^ CFU) 500 ml/day	Conventional therapy alone (no placebo)	For duration of hospital admission or patient request to stop	Mortality: No significant impact on mortality Mortality rate in both groups 35%	Very low
Nomura et al. [35]	Control: Median age: 69 [50–88] Intervention: Median age: 66 [30–83]	64	Patients scheduled for pancreaticoduodenectomy	Surgical centre, Japan	RCT	PROBIOTIC BIO-THREE^®^ Tablets *Enterococcus faecalis *T-110 2 mg,* Clostridium butyricum *TO-A 10 mg,* Bacillus mesentericus *TO-A 10 mg Six tablets/day	Nil	Immediately after admission (3–15 days pre-operatively), restarted on second day post operatively and continued to discharge	Mortality: Numbers too small to draw conclusions on mortality Only one death in study group (in control group due to abdominal bleeding) Length of stay: Reduced postoperative LOS in intervention group Median (range) days: 19 (11–40) vs. 24 (11–91) (*p* = 0.04)	Very low
Rongrungruang et al. [33]	Control: 68.9 (± 18.4) [20–97] Intervention: 73.1 (± 13.2) [30–94]	150	Ventilated patients	Intensive Care Unit, Thailand	RCT	PROBIOTIC + standard care *Lactobacillus casei*—Yakult (8 × 10^9^ CFU) 80 ml for oral care (after standard care) and additional 80 ml via enteral feeding OD	Standard care (chlorhexidine 2% oral care QDS)	28 days or until ETT removed	Length of stay: No significant difference in LOS Median (range) days: 20 (2–106) vs. 19 (3–171) (*p* = 0.79) Mortality: No significant difference in mortality Day 28: 24% vs. 22.7% (*p* = 0.85) Day 90: 33.3% vs. 34.7% (*p* = 0.86)	Very low
Shinkai et al. [16]	Control: 70.9 (± 3.8) Intervention 1: (low dose) 71.0 (± 4.0) Intervention 2: (high dose) 70.8 (± 3.4)	278	Community dwelling older adults (> 65 years)	Community, Japan	RCT	PROBIOTIC Heat killed* Lactobacillus pentosus *b240 Low dose: 2 × 10^9^ cells High dose: 2 × 10^10^ cells One tablet/day at breakfast	One tablet/day at breakfast	20 weeks	Mood: No improvement in mood with intervention Mental health, emotional role or social functioning subscales of SF-36 unchanged with intervention General health perception: 55.5(± 7.8) to 56.6 (± 8.2) vs. 54.9 (± 7.8) to 53.4 (± 8.6) Physical function: No improvement in physical function Self-reported physical functioning or physical role subscales of SF-36 not changed by intervention	Moderate
Tamtaji et al. [28]	Control: 78.8 (± 10.2) Intervention: 76.2 (± 8.1)	79	Older adults with a diagnosis of AD	Community, Iran	RCT	PROBIOTIC + Selenium *Lactobacillus acidophilus, Bifidobacterium bifidum, Bifidobacterium longum *(2 × 10^9^ CFU/day of each) + selenium 200 ng/day	Selenium (exclude placebo from SR as not single intervention)	12 weeks	Cognition: Improved cognition with intervention MMSE: 1.5(± 1.3) vs 0.5(± 1.2) (*p* < 0.001)	Moderate
Buigues et al. [17]	Control: 73.4 (± 1.8) Intervention: 74.2 (± 1.6)	50	Mobile, non-demented nursing home residents	Nursing homes, Spain	RCT	PREBIOTIC Darmocare Pre^®^ (inulin min. 3375 mg, plus fructooligosaccharides min. 3488 mg per level measuring spoon of 7.5 g) After breakfast daily	Placebo (maltodextrin)	13 weeks	Frailty: Overall no significant effect on frailty Improved exhaustion Mean frailty score (fried): Intervention: 2.8 (± 1.0) at baseline to 2.5 (± 0.8) after intervention Exhaustion (self-reported 0–3): 1.4 (± 1.7) to 0.8 (± 0.4) vs. 1.1 (± 1.7) to 1.7 (± 1.2) (*p* = 0.002) Cognition: No significant effect on MMSE MMSE: 26.5 (± 3.1) to 26.4 (± 2.2) vs. 26.1 (± 2.2) to 25.9 (± 2.1) (*p* = 0.85) Physical function: Improved hand grip strength No significant difference in ADLs (Barthel) or walking speed R hand grip strength: 10.6 (± 8.2) to 12.4 (± 3.2) vs. 11.5 (± 5.7) to 12.4 (± 3.2) (*p* < 0.05) Barthel index: 74.6 (± 17.7) to 77.1 (± 29.9) vs. 76.2 (± 13.0) to 78.3 (± 13.9) (*p* = 0.87) Walking speed (time needed to walk 4.6 m): 8.4 (± 6.0) to 7.9 (± 4.5) vs. 8.6 (± 9.0) to 8.7 (± 4.2) (*p* = 0.48)	Low
Lewis et al. [31]	Control: Median age: 74 [IQR: 63–80] Intervention: Median age: 76 [IQR: 65–84)	142	Consecutive inpatients with *Clostridium difficile* toxin assoc. diarrhoea	Hospital, UK	RCT	PREBIOTIC + standard treatment Oligofructose 12 g/day	Placebo (sucrose 12 g/day) + standard treatment	Started as soon as possible after dx and taken for 30 days after cessation of diarrhoea	Mortality: No obvious significant difference in mortality demonstrated Died: 9 (13%) vs. 10 (14%) Length of stay: No obvious significant effect on LOS Median: 27 [IQR: 16–51] vs. Median: 29 [IQR: 14–58]	Low
Theou et al. [22]	Control: 75.9 (± 7.8) Intervention: 74.9 (± 6.9)	50	Mobile, non-demented nursing home residents	Nursing homes, Spain	RCT 2ry analysis of [26]	PREBIOTIC Darmocare Pre^®^ (inulin min. 3375 mg, plus fructooligosaccharides min. 3488 mg per level measuring spoon of 7.5 g) 7.5 g after breakfast daily	Placebo (maltodextrin)	13 weeks	Frailty: Intervention reduced frailty compared to placebo Significant interaction (*p* < 0.001) of time with the treatment group for FI 13 weeks follow up: Intervention group had lower FI levels compared to baseline (*p* < 0.001) vs. placebo had higher FI levels (*p* = 0.012) Average reduction of 0.02 (± 0.02) in intervention and increase of 0.01 (± 0.01) FI in placebo Moderately/severely frail group had more significant improvement	Moderate
Jain et al. [30]	Control: Median age: 73 [IQR: 65–80] Intervention: Median age: 72 [IQR: 62–77]	90	ITU and HDU pts admitted for < 24 h No specific age range	Intensive Care Unit, UK	RCT	SYNBIOTIC Trevis™ capsules: *Lactobacillus acidophilus *La5,* Bifidobacterium lactis *Bb12,* Streptococcus thermophilus* and *Lactobacillus bulgaricus *(4 × 10^9^ CFU of each) Raftilose™ powder, Orafti Active Food ingredients: Oligofructose One Trevis™ capsule TDS 7.5 g Raftilose™ BD	Placebo capsules and powdered sucrose	Until death or discharge from current hospital	Mortality: No significant difference in mortality 49% vs. 45% (*p* = 0.672) Length of stay: No significant difference in LOS Median LOS 15 days [IQR: 9–26] vs. 14 days [IQR: 9–29] (*p* = 0.913)	Low
Louzada et al. [27]	Control: 77.0 (± 1.3) Intervention: 77.2 (± 1.3)	49	Older persons (65–90). Pre-frail individuals registered with single health centre	Community, Brazil	RCT- 2ry analysis	SYNBIOTIC *Lactobacillus paracasei, Lactobacillus ramnosus, Lactobaciullus acidophilus, Bifidobacterium lactis *(10^8^–10^9^ CFU of each) Fructooligosaccharide (6 g) Two daily doses (6 g + 6 g)	Placebo (6 g + 6 g maltodextrin)	24 weeks	Cognition and mood: No significant effect on mood or cognition was found MMSE: median 25.0 to 25.9 vs. median 27.0 to 26.5 (*p* = 0.25) GDS-15: median 3.0 to 3.0 vs 2.5 to 3 (*p* = 0.47)	Low
Neto et al. [18]	All participants: 67.9 (± 4.5) [60–74]	17	Community dwelling older adults fulfilling one of Fried’s frailty criteria	Community, Brazil	RCT	SYNBIOTIC *Lactobacillus paracasei, Lactobacillus ramnosus, Lactobaciullus acidophilus, Bifidobacterium lactis *(10^8^–10^9^ CFU of each) Fructooligosaccharide (6 g) Once daily dose diluted in water to be drank following the last meal of the day	Placebo (maltodextrin)	3 months	Physical function: No significant impact on grip strength Grip strength (*N*): 15.0 (± 5.2) to 15.7 (± 5.3) vs. 15.9 (± 2.7) to 17.2 (± 3.9)	Very low
Östlund-Lagerström et al. [19]	Control: 72.6 (± 5.8) Intervention: 72 (± 5.6)	249	Free-living older adults living in their own homes	Community, Sweden	RCT	SYNBIOTIC (although note study refers to PROBIOTIC only) *Lactobacillus reuteri *DSM17938 (1 × 10^8^ CFU/stick pack), Rhamnose, galactooligosaccharide and maltodextrin To a total weight of 1 g	Placebo (maltodextrin)	12 weeks	Mood: No significant differences in anxiety or depression scores HADS: − 0.32 (± 3.5) to − 0.18 (± 4.4) vs. − 0.41 (± 2.9) to 0.005 (± 5.2) (*p* = 0.89) PSS: − 0.41 (± 4.9) to − 1.55 (± 4.8) vs. − 0.24 (± 5.4) to − 1.12 (± 4.4) (*p* = 0.57) Physical function: No significant difference in scores looking at functional wellbeing EQ-5D-5L: Index: 0 (± 0.1) to 0 (± 0.1) vs − 0.01 (± 0.1) to 0 (± 0.1) (*p* = 0.87) VAS: 1.89 (± 10.7) to 2.88 (± 9.8) vs. 0.60 (± 11.5) to 3.47 (± 7.7) (*p* = 0.66)	Moderate
Shimizu et al. [34]	Control: Median age: 74 [IQR: 64–81] Intervention: Median age: 74 [IQR: 64–82]	77	Septic patients placed on a ventilator within 3 days of ITU admission	Intensive Care Unit, Japan	RCT	SYNBIOTIC Yakult BL Seichoyaku 1 × 10^8^* Bifidobacterium Breve *strain Yakult/g, 1 × 10^8^* Lactobacillus casei *strain Shirota/g Galactooligosaccharides (Oligomate S-HP, Yakult Honsha) Yakult BL Seichoyaku 3 g/day + galactooligosaccharides 10 g/day	Standard care	Until oral intake initiated	Mortality: No significant difference in mortality seen 10.8% vs. 8.6%% (*p* = 0.84)	Very low

*RCT* randomised control trial, *CFU* colony-forming units, *IQR* inter-quartile range, *ADLs* activities of daily living, *LOS* length of stay, *FI* frailty index, *HADS* Hospital Anxiety and Depression Score, *PSS* Perceived Stress Score, *VAS* Visual Analogue Scale

### Physical function

Five studies evaluated physical function [16–20] and found minimal change in outcomes with PPS. Although our search included falls as a marker of physical function, no studies were identified that included falls as an outcome.

Buigues et al. had the only statistically significant finding of these studies (GRADE certainty = ‘low’). They conducted a small (*n* = 50) RCT looking at the impact of prebiotics on frailty in a nursing home population over 13 weeks. The overall findings on frailty syndrome were inconclusive (see below) but they did demonstrate improved grip strength and reduced self-reported exhaustion in the intervention group compared to placebo [right hand grip strength (kg): 10.6 ± 8.2 to 12.4 ± 3.2 vs. 11.5 ± 5.7 to 12.4 ± 3.2 (*p* < 0.05)]. Exhaustion was assessed using a patient questionnaire with a scale of 0–3 to reflect frequency of feeling exhausted across the week. The improvement in grip strength was significant in the right hand only (*p* = 0.50 on the left). There was no improvement in activities of daily living (ADLs) measured using the Barthel index and no improvement in walking speed.

Neto et al. (GRADE certainty = ‘very low’) conducted a small pilot study (*n* = 17) evaluating the effect of synbiotic intake daily over 3 months on inflammation and body composition in older people [18]. One of the outcome measures was grip strength. No significant effect of synbiotic intake on grip strength was demonstrated although the results were limited by the small sample size and a poorly matched control group.

Östlund-Lagerström et al. (GRADE certainty = ‘moderate’) conduced a double-blinded RCT examining the effect of a probiotic on digestive health and wellbeing in older adults [19]. Although the study intervention is described by the authors as being a probiotic, the probiotic was combined with a galacto-oligosaccharide and would, therefore, be defined as a synbiotic. The intervention was given to the study population (*n* = 249) for 12 weeks. The primary outcome measures were gastrointestinal effects but secondary measures included assessment of quality of life using an EQ-5D-5L questionnaire which looks at mobility, self-care and ADLs alongside wellbeing [21]. No significant differences in these markers of physical function were seen between the placebo and intervention group although this is an atypical method of assessing physical function.

Shinkai et al. (GRADE certainty = ‘moderate’) conducted a double-blinded RCT primarily evaluating the dose-dependent effect of a probiotic on common cold incidence in 278 older adults over 20 weeks [16]. They assessed quality of life as a secondary outcome using the SF-36v2™ questionnaire. One of the subscales of this assessment is self-reported physical functioning. No significant difference in physical function was reported in either the low- or high-dose intervention group compared to placebo.

Mañé et al. (GRADE certainty = ‘low’) conducted a double-blinded RCT primarily looking at the effect of two different strengths of probiotic on immunity in older people (*n* = 50) over 12 weeks. They assessed ADLs as a secondary outcome by recording the Barthel index at baseline and after 12 weeks and 24 weeks of receiving a probiotic. They found no significant change in either the intervention group or the placebo group [20].

### Frailty

Only two papers evaluated the effect of PPS on frailty and both of these were based on the same RCT by Buigues et al. [17, 22]. They reached differing conclusions using different methods to assess frailty. The authors of the original study reported that the prebiotic intervention had no impact on frailty using a phenotypic model (fried frailty criteria [23]). Theou et al. (GRADE certainty = ‘moderate’) postulated that using a frailty index (FI) would be a better tool to assess impact as it would include items from more domains. The authors constructed their own FI by combining features of the Geriatric Depression Scale, Athens Insomnia Scale, Tinetti Balance and Gait Evaluation, Norton Scale for Assessing Risk of Pressure Ulcers, The Barthel Index, Mini-Mental State Examination (MMSE), comorbidities and medication. They used this tool to create a continuous score of 0–1 and then categorised participants into non-frail, mildly frail and moderately/severely frail based on their score. They estimated that a change over the 13 weeks trial of 0.03 on this FI would be considered as a meaningful change. Using this alternative method of assessing frailty, they did demonstrate a significantly greater improvement in frailty in the prebiotic group compared to placebo over time although the change in the group as a whole did not meet their own criteria for a ‘meaningful’ change. The improvement was most marked in the moderately/severely frail group who were the only subgroup who demonstrated a ‘meaningful’ change in FI as defined by the study design (0.04 ± 0.01). Baseline FI in all subgroups collated ranged from 0.22 ± 0.09 to 0.20 ± 0.08 in the intervention group vs. 0.23 ± 0.11 to 0.24 ± 0.12 in the placebo group.

### Cognition and mood

Eight studies evaluated the outcomes of mood and cognition [16, 17, 19, 24–28]. Five of these used a probiotic intervention, one used a prebiotic and two a synbiotic. Four of these studies (all using a probiotic) reported a positive effect on cognition in participants with pre-existing cognitive impairment. None reported a positive effect on mood. Four studies looked at cognition alone, two at cognition and mood and two assessed mood alone.

Akbari et al. (GRADE certainty = ‘low’) reported the most significant finding on cognition of these collected studies. They conducted a double-blinded RCT involving 60 community dwelling participants with Alzheimer’s disease (AD) randomised to have an intervention consisting of four probiotic strains of bacteria with milk each day or to have just milk alone for 12 weeks [24]. The results showed a significant improvement in MMSE in the intervention group [27.90% ± 8.07 vs.  − 5.03% ± 3.00 (*p* < 0.001)].

Hwang et al. (GRADE certainty = ‘low’) conducted an RCT looking at the effects of a probiotic on cognition in participants (*n* = 100) with mild cognitive impairment (MCI) [25]. The authors used a battery of neuro-cognitive tests to assess cognition at baseline and after 12 weeks of either placebo or a probiotic. They found a greater improvement in combined cognitive function in the intervention group than the placebo group (*p* = 0.02) although scores in both groups improved over time. By breaking down the assessment into different fields (attention, working memory and verbal memory), a significant improvement in the field of attention specifically was demonstrated in the intervention group. There was no significant improvement in the fields of working memory and verbal memory.

Tamtaji et al. (GRADE certainty = ‘moderate’) conducted a double-blinded RCT looking at the clinical, metabolic and genetic effects of probiotic and selenium on participants (*n* = 79) with AD. The intervention was given for 12 weeks.[28]. As this review is limited to look at a single intervention only, we considered the effect of probiotic and selenium vs. selenium alone and did not compare with the placebo group. This study demonstrated a statistically significant improvement in cognition (as measured by MMSE) in the probiotic and selenium group compared to selenium alone. The change in score was 1.5 ± 1.3 vs. 0.5 ± 1.2 (*p* < 0.001). This change is just above the minimum clinically important difference for MMSE of 1.4 points [29].

Buigues et al.’s study using a prebiotic intervention (discussed in previous parameters) looked at cognition within the context of assessing frailty and did not demonstrate any effect of the prebiotic intervention on MMSE [17].

Kobayashi et al. (GRADE certainty = ‘very low’) conducted an open-label single-arm study evaluating the effect of probiotic supplementation on cognitive decline in older adults with MCI [26]. They reported improved cognitive function after probiotic supplementation with resolution of MCI in 13 of 19 cases (68%) by 24 weeks. This study had significant limitations due to its size and lack of control group. Although only 19 of the 27 individuals originally recruited completed the study (70%), all participants’ data were included in the baseline characteristics which were then contrasted with the final characteristics of just those who completed the study. The digit symbol substitution test (DSST) was also used as an assessment of cognition and no significant difference was seen in DSST scores over time. The study also assessed mood using the Profile of Mood 2nd Edition (POMS2) score. No significant change in total mood disturbance (calculated using POMS2) was demonstrated.

Louzada et al. (GRADE certainty = ‘low’) conducted a secondary data analysis of an RCT which was originally designed to look at the effect of synbiotics on systematic inflammation and lean body mass. The intervention was given for 24 weeks. This secondary study aimed to look at systemic inflammation and symptoms of brain disorders [27]. Older, community dwelling volunteers with at least one characteristic from Fried’s frailty criteria [23] were recruited to the original study (*n* = 49). The secondary data analysis found no difference in mood after 6 months between the intervention and placebo group (assessed using Geriatric Depression Scale-15) and no difference in cognition (assessed using MMSE) between groups. MMSE improved slightly across both groups over time which is likely to represent learning effect.

Shinkai et al. (discussed above) measured secondary outcomes of quality of life (using the SF-36v2™ questionnaire) and assessed mood using the Profile of Mood States (POMS) questionnaire [16]. A probiotic intervention was used. The subscales of the SF-36 include ‘Role emotional’, ‘Social Functioning’ and ‘Mental Health’ which are all markers of mood. No change in these outcomes was demonstrated with either low- or high-dose probiotic intervention. No significant difference was seen in the POMS results between the different groups either. The only positive finding from quality-of-life assessment was improved health perception in the higher-dose probiotic group. This finding is not relevant to this review as ‘health perception’ is not a surrogate marker of mood alone.

Östlund-Lagerström et al. (discussed above) also looked at mood outcomes in their RCT. They found no effect of a synbiotic on mood with no significant change in the Hospital Anxiety and Depression Score (HADS) or the Perceived Stress Score [19].

### Mortality

Seven of the identified studies recorded mortality as an outcome measure (all as secondary outcomes) [20, 30–35]. Four evaluated probiotics as the intervention, one prebiotic and two synbiotics.

Only one study found any impact of PPS on mortality reporting a trend towards reduced mortality but the study was underpowered [20]. The remaining six studies found no significant difference in mortality in patients receiving PPS. Four of the studies were assessed as having ‘very low’ GRADE certainty for this review and the remaining two were assessed as ‘low’.

### Length of stay

Five studies reported receipt of care (all as length of hospital admission) as a secondary outcome [30, 31, 33, 35, 36]. Two studies reported a significantly shorter length of stay.

Kotzampassi et al. (GRADE certainty = ‘very low’) conducted an RCT evaluating the effect of a four-probiotic regimen given for 14 days on postoperative complications after colorectal surgery (*n* = 168) [36]. Recruitment was not limited to older people, but the mean age of their participants was greater than 65 years. The participants receiving a probiotic preoperatively and then for the subsequent 14 days had a shorter length of stay compared to those receiving a placebo (log-rank 20.3, *p* < 0.0001). The median time until discharge from hospital was 8 days in the probiotic group compared to 10 days in the placebo group.

Nomura et al. (GRADE certainty = ‘very low’) conducted an unblinded RCT using a probiotic tablet containing three bacteria (*n* = 64) [35]. Patients scheduled to undergo pancreatectomy were randomised to receive the probiotic perioperatively until discharge home or to the control group which received standard care. They report a reduced postoperative length of stay (median 19 days vs. 24 days, *p* = 0.04) in patients receiving the probiotic.

The other three studies reported no significant effect of PPS intervention vs. placebo on length of hospital admission. These studies were all assessed as having ‘low’ or ‘very low’ certainty of evidence according to GRADE criteria. One of these studies was an RCT of a probiotic given to ventilated patients for 28 days or until they were extubated [33]. Median length of stay was 20 days in the intervention group (range 2–106) vs. 19 days in the control group (range 3–171) (*p* = 0.79). Lewis et al. used a prebiotic intervention for patients with clostridium associated diarrhoea [31]. Median length of stay was 27 days in the intervention group vs. 29 in the placebo group. The final study used a synbiotic intervention on patients in high dependency or intensive care [30]. The authors report a median length of stay of 15 days (interquartile range 9–26) in the intervention group vs 14 days (interquartile range 9–29) in the placebo group.

No papers reported the outcome measure of number of hospital admissions.

### Adverse effects

Few adverse effects were reported in these studies. Buigues et al. did report a significant difference in dropout rate between the prebiotic and control group with 29% of participants (8/28) in the intervention group stopping the treatment compared to only 9% (2/22) of the placebo group. Cramps and diarrhoea were among the reasons given although cramps were also cited by a participant from the placebo group. The authors report that flatulence and cramping occurred in about 30% of the intervention group and diarrhoea in 4% but that the majority of symptoms resolved within 8 days of starting the prebiotic and were generally well tolerated (the participants who dropped out did so before 8 days) [17]. The remaining studies did not demonstrate any clear difference in adverse events between the intervention and control groups. Adverse events reported by Hwang et al. included dizziness, stomach aches, headaches, gastritis, erectile dysfunction and seborrheic dermatitis in the intervention group receiving probiotic capsules daily for 12 weeks [14% of participants (7/50) in the intervention group reported an adverse event]. Irregular bowel movements, stomach aches and erectile dysfunction were the adverse events reported in the placebo group [10% of participants in the placebo group (5/50) reported an adverse event] [25]. More patients dropped out of the placebo group than the probiotic intervention group in Östlund-Lagerström et al.’s study (20 vs. 11) with no serious adverse events in either group [19].

## Discussion

This review identified published studies that evaluated the impact of PPS on functional outcomes amongst older adults. There is limited evidence of the impact of PPS on physical function as only one of five studies reported benefit (in right-handed grip strength and self-reported exhaustion with a prebiotic intervention). The impact of prebiotics on frailty is mixed depending on the frailty tool used (no studies were found that used a probiotic or synbiotic). There is some evidence that probiotics can improve cognition but not mood. Four of six studies reported benefit in cognition scores in participants with AD and MCI receiving probiotics (although one of these studies had significant methodological limitations). The impact of PPS on mortality is unclear as only one study (out of seven) showed a significant impact and this had a small sample size. Similarly, the impact on length of stay appears weak with only two studies of mixed ages reporting a benefit after surgery. Few adverse outcomes were reported in these papers and so PPS does appear to be a safe option for further investigation.

Only one study identified any benefit on physical function in spite of growing evidence of a ‘gut-muscle’ axis with gut composition affecting muscle mass [37] and animal studies showing improved muscle strength and function in aged rodents receiving probiotics [38]. No change in body composition or BMI was seen in older individuals given a probiotic [24], prebiotic [17] or synbiotic [18] in the studies we identified although the intervention periods were all of a relatively short duration (maximum 3 months). Reduced risk of falls has been demonstrated in younger, cognitively impaired cirrhotic patients taking a probiotic [39] but no studies evaluating the effect of PPS on falls in older people were identified in this review.

It is not clear whether findings from animal studies and studies of a younger population have not been replicated in older adults due to differences in the ageing microbiome, age-related changes, such as frailty or sarcopenia, or whether it is due to a lack of research in this area to date. There were methodological limitations to the studies we identified evaluating physical function. One study was particularly limited by its very small sample size and poorly matched control group [18] and another only considered self-reported physical function as one subset of a questionnaire on quality of life [16]. Three of the five studies only included physical function as a secondary outcome [16, 19, 20]. Östlund-Lagerström et al. debated whether a lack of significant effect may be due to using too low a dose of probiotic or the inclusion of participants who were taking a proton pump inhibitor [19]. Older adults are more likely to be taking medications or suffer co-morbidities which could alter the outcomes of PPS as an intervention. These important potential limitations in studying the older population apply to all of the outcomes reported in this review.

Frailty is an important functional marker for older individuals and for healthcare providers. As in clinical practice, one of the issues within research is how best to assess frailty. There is no consensus on how best to measure/identify frailty. This issue has been clearly demonstrated with our finding of two differing conclusions being reached from the same study data through using different frailty assessment tools [17, 22]. The only study identified in this review evaluating frailty was very small (only 50 participants completed the study) and the secondary analysis of this RCT used an unvalidated derived FI. We are unable to make any conclusions on the potential effects of PPS on frailty due to the lack of studies in this field.

Within this review, eight studies reported on mood and cognition. Animal studies support the theory of gut–brain regulation of cognitive symptoms [40] and have indicated that synbiotic supplementation could help counteract age-related memory loss [41] and that gut microbiota changes are associated with social avoidance and depression in mice [42]. A 2018 systematic review summarizing the literature on the gut microbiota alterations associated with cognitive frailty, MCI and dementia and evaluating the effects of prebiotics or probiotics on cognitive symptoms in animal models of aging and on human beings found inadequate human data to make any recommendations [43]. A literature review evaluating the impact of prebiotics on brain function in older adults reported that prebiotics improve cognitive function, reaction time and mood and decrease anxiety in healthy young and middle-aged adults but there was a lack of studies looking specifically at an older population (only one study identified in the literature review evaluated older participants) [44]. A systematic review published in 2016 looking at the effects of probiotics on depression concluded a positive effect but four of five studies only included young/middle-aged participants [45]. A study looking at the potential effects of altering the microbiome through probiotics and synbiotics found that they appear to have a positive impact on the human microbiome via gut–brain axis and the authors concluded that this translates to the potential to decrease the risk of neurodegenerative diseases [46] although this impact was not directly studied. Our review has shown that there is limited evidence that PPS could improve cognition but not mood in older people. Of note, several studies have shown no impact on mood and cognition and one study looking at a non-age selected population (average age 61) found an incidental finding of poorer performance on two measures of memory in participants taking probiotics [47].

Three studies in this review identified an improvement in MMSE in older people who had been taking a probiotic [24, 26, 28] and a fourth study reported improved combined cognitive function after probiotics using a neuro-cognitive battery test [25]. All of these studies involved participants with pre-existing cognitive impairment. One of these studies was of poor quality with no control group and comparison of all 27 participants enrolled at baseline with the 19 participants who completed the study [26]. If participants with worse cognitive function were more likely to drop out this would bias the results. There is a recognised learned effect of repeated MMSE testing, so without a control group, it is difficult to conclude that a marginally improved MMSE represents a significant result. Another of these studies found only a very minimal improvement in MMSE [28] and the standardisation of the neuro-cognitive assessment in one makes the data difficult to interpret [25]. All of these studies used different combinations and concentrations of probiotics and one of the studies co-supplemented with selenium resulting in considerable heterogeneity in the interventions.

We did not identify any studies evaluating mortality or length of hospitalisation as primary outcomes in older people taking PPS. Although they are reported as secondary outcomes in a number of studies (mostly looking at perioperative or critical care patients), the studies were typically underpowered for these outcome measures. Studies reporting length of hospital admission among younger patients have also largely focused on patients in intensive care and results have been mixed [48, 49]. Two studies did find a reduction in length of hospital admission in an intervention group taking probiotics. Both of these studies have limitations regarding our review question because of the wide age range of the samples [35, 36] and one study sample was small with no blinding [35].

Prebiotics occur naturally in many food items which is a confounding factor for clinical trials. Diet itself has a huge impact on the microbiome aside from the impact of prebiotic food. Although eight of these 18 studies recorded dietary intake in some form, only one has accounted for dietary variation in their results [16]. Conducting a trial where all participants have a regulated diet would be very difficult. This further emphasises the need for studies in this field to measure and adjust for dietary differences.

This systematic review conformed to PRISMA guidance and was performed with the assistance of the healthcare library services at the University of Southampton. Most steps have been independently performed by two reviewers working in tandem to limit bias. Quality appraisal was performed using the GRADE certainty method.

There are limitations to this study including heterogenous interventions, the predominance of secondary outcome measures and small sample sizes in the identified studies. The inter-library loan services were unable to retrieve three full texts which may have been eligible for this review. One further study was excluded as we were unable to access the full text in English [50]. In the English language, abstract of this study, Lopez et al. report that adults (median age 69 years) with multiorgan failure who took synbiotics had no difference in length of stay or mortality compared to the placebo group (recorded as secondary outcomes). Finally, the GRADE certainty method was used to appraise the quality of the identified studies. This method scores the certainty of the evidence specific to our review question and so is not a measure of the quality of the studies as a whole. Fourteen of the included studies were assessed as low or very low certainty of evidence for our question which is a significant limitation and reflects the prevalence of secondary outcome measures being evaluated.

## Conclusion

This review identified little evidence of benefit on physical function, frailty, mood, mortality or length of hospital admission from the use of PPS among older people. There was limited evidence for improved cognition with use of probiotics in those with pre-existing cognitive impairment. However, a lack of evidence from a small number of heterogenous studies does not mean that there is lack of effect. Current evidence around the use of PPS focuses largely on laboratory markers and there are few studies focused specifically on the older population. Robust research with larger studies, consistency of interventions and clear assessment of confounding variables (such as diet, co-morbidities and medications) is needed to evaluate the effect of PPS on functional outcomes in an older population. There is currently inadequate evidence for recommending PPS use to older people in general.

## Electronic supplementary material

Below is the link to the electronic supplementary material.

Supplementary file1 (PDF 101 kb)
