# Supplementary material for: Can probiotics, prebiotics and synbiotics improve functional outcomes for older people: a systematic review
Source: Eur Geriatr Med. 2020 Sep 24;11(6):975–93. doi: 10.1007/s41999-020-00396-x (PMC7515554; doi:10.1007/s41999-020-00396-x)
Supplement: Supplementary file 1 — Supplementary file1 (PDF 101 kb) [file 41999_2020_396_MOESM1_ESM.pdf]

Article Title: Can probiotics, prebiotics and synbiotics improve functional outcomes for older people: a systematic review

Journal Name: European Geriatric Medicine

Names of authors: Coutts L, Ibrahim K, Tan QY, Lim SER, Cox NJ and Roberts HC

Affiliation and addresses of authors: Academic Geriatric Medicine, University of Southampton, Southampton General Hospital mailpoint 807, Southampton, SO16 6YD, UK

E-mail address of corresponding author: l.coutts@soton.ac.uk

Appendix 1: Search Strategy

HDAS Systematic Review Search:

- Medline
- EMBASE
- CINAHL
- Pubmed

- All- title and abstract
- Limits: Year of publication- no earlier than 2000

Spine search terms:

1. Older*
2. Elder*
3. Aged
4. Geriatric
5. Dementia
6. Dementia thesaurus
7. “Parkinson’s Disease”
8. 1 OR 2 OR 3 OR 4 OR 6 OR 7
9. Prebiotic*
10. Prebiotic in thesaurus
11. Probiotic*
12. Probiotic in thesaurus
13. Synbiotic*
14. Synbiotic in thesaurus
15. 9 OR 10 OR 11 OR 12 OR 13 OR 14

Physical function:

16. Fall*
17. Fall thesaurus
18. “Activities of Daily Living”
19. ADL thesaurus
20. Strength
21. Strength thesaurus
22. Physical ADJ2 Function
23. 1 OR 2 OR 3 OR 4 OR 5 OR 6 OR 7

Frailty:

24. Frail*

Mood and Cognition:

25. Cogniti*
26. Cognition thesaurus
27. Mood
28. Mood thesaurus
29. Depress*
30. Depression thesaurus
31. 1 OR 2 OR 3 OR 4 OR 5 OR 6

Mortality:

32. Mortality
33. Death

34. 32 OR 33

Receipt of care- hospital admissions or length of stay

35. Hospital ADJ2 Admission*
36. “Length of stay”
37. 35 OR 26

Final result:

38. 23 OR 24 OR 31 OR 34 OR 37
39. 8 AND 15 AND 38 [2000 – 2020]
